# Reviews and Book Notices

**Published:** 1879-06

**Authors:** 


### Reviews and Book Notices.

ARTICLE XVIII.—PRINCIPLES AND PRACTICE OF GYNÆCOLOGY, FOR THE USE OF STUDENTS AND PRACTITIONERS OF MEDICINE. In one large and very handsome octavo volume of 853 pages, with 130 illustrations. By Thomas Addis Emmet, Surgeon to the Woman's Hospital in the State of New York. Henry C. Lee, 1879.

Thirty years have scarcely passed away since one or two lectures on the diseases peculiar to women comprehended all that the most eminent teachers had to offer. But within these two or three decades various types of vaginal specula have appeared from week to week, with singular regularity. Many of these instruments, by spreading the fame or stimulating the pride of their inventors, rapidly fulfilled the essential part of their mission. It was with Sims' speculum and the lateral method of speculum examination that gynæcology received its great impulse. We are advocates of the opinion expressed in the second chapter of this book, that "in a single generation the use of this instrument has advanced the knowledge and treatment of the diseases, and especially the injuries of woman, from profound ignorance to a front rank, if indeed, not beyond that of any other branch of surgery." When the way had been opened by Sims' and other specula, for the more intelligent study of the reproductive organs of woman, a period of experiment followed and of this period modern gynæcology is the logical result. But respecting uterine pathology and theraputics the mass of the profession is yet in unstable equilibrium. The literature of the subject, pregnant with opinions and replete with promises, contains much that is uncrystallized and unsatisfactory. The enthusiasm of the last generation has produced material in abundance, all of which is admirably compiled but not sifted. The profession of the whole world, therefore, has awaited with no ordinary anticipation and now receives with distinguished welcome, an author whose ability and experience qualify him to comprehend the subject in an original work containing what he knows, not what others have said. Such a work is now under review. A work almost as remarkable for the error which it omits or condemns as for the truth which it contains.

For many years the Woman's Hospital, in the State of New York, was the only institution of its special character in the world, and for this reason it afforded unparalleled advantages for continued observation of individual cases, and therefore for testing the merit of the many brilliant operations which had been introuced and which have marked the progress of this American specialty.

Dr. Emmet has been connected with this institution since its opening in 1854, and the history of his entire service includes a large majority of its patients and surgical operations. To these clinical advantages have been added those of his "private hospital and of an extensive consulting practice." His material, therefore, for original observation and generalization has been exceptional and unequaled. His previous literary contributions though not voluminous show ample evidence of honest and careful observation, of original, conservative and logical thought.

From the preface may be quoted the following detached sentences:

"This work is essentially a clinical digest. It includes the results of my individual experience and aims to represent the actual state of gynæcological science and art. * * * With the exception of two plates taken from Savage's work, and some of the instruments, all the illustrations are original, the drawings having been furnished by myself. In attempting to ascertain and formulate the laws which apply to disease and to analyze the results of treatment, I have compressed numerous histories into a number of statistical tables which present in brief space information that hundreds of pages would scarcely have sufficed to contain in detail. Their parallel, it is believed, is not to be found in the whole range of gynæcological literature.

*Chapter I, on the Relations of Climate, Education and Social Conditions to Development*, fully indorses the views of the late

Dr. Edward H. Clarke,* of Boston, and claims that the influence of our American climate, and our educational and social systems is "even more serious than he has represented." If this chapter be an exaggeration its error is certainly on the safe side.

*Chapters II and III* are given to a consideration of the instruments usually found in a complete gynæcological case, and are profusely illustrated by engravings. It is noticable that these instruments, with few exceptions, bear the name either of Emmet or Sims. To one or two paragraphs we invite special attention:

> "As long as the sole use of the speculum was to bring the cervix into view and to facilitate the passage of the porte-caustique, in the treatment of a supposed ulceration, the cylindrical speculum sufficed. With the advance of knowledge in the treatment of uterine disease it became necessary to gain more space and light, and the cylindrical speculum has gradually been superceded by various instruments with expanding blades to open the upper portion of the vagina. * * * I have known both retroversion and prolapse to occur from repeated use of the valvular speculum, which had stretched the anterior wall of the vagina. The amount of light and space obtained by any of these instruments is very small compared with what is afforded by Sims', and they are useless for all surgical purposes. Full justice, in the light of our present knowledge, cannot be done in the treatment of uterine disease by any other instrument than this perineal retractor, or some other based upon the same principle and like it capable of exposing the whole vagina.

This chapter contains a description of the author's method of applying the vaginal tampon for uterine hæmorrhage, a method superior to all others.

*Chapter IV: Physical Examination of the Female Pelvic Organs. Form for record of cases.* The dangers of untimely and unskillful exploration of the uterus, especially when old or recent cellulitis be present are clearly indicated. Profoundly impressed with the importance of this caution, the author repeats it many times in the remaining chapters. The young practitioner and student will appreciate the minute and full directions here given for the accurate diagnosis of diseases. Simpson's sound is condemned as a dangerous instrument and the fine silver probe is substituted as safer and in the skillful hands more efficient.

*Sex in Education, or a fair chance for the Girls, Boston, 1873. The Building of a Brain, Boston, 1874.

*Chapters V and VI are given to the Causes of Diseases Reflex and Direct and to the Principles of General Treatment.*

*Chapters VII and VIII on Local Treatment* deplore the lack of method which has always characterized the madness of good doctors in local applications to the uterus. We fully commend all that is said of the hot water vaginal douche, since in our own experience this agent alone has proved more useful than all other local treatment combined. The following is important:

> "No plan of treatment could be more rational, or appeal more forcibly to the good judgment of every one. But, unfortunately from a neglect of details, it is rare that the slightest benefit is derived from the use of these injections, although so many years have elapsed since the profession has been fully informed as to their action."

The author then explains his own method of applying the douche and points out the causes of failure in others.

The actual cautery, the mineral acids, nitrate of silver and all escharotics are justly condemned as productive of more evil than good. The author says:

> "If the so-called ulceration of the cervix be accepted as a cause and not an effect, the use of the caustic is consistent practice, and should be persevered in until the surface is healed.
>
> But if it be held that the increased secretion is simply an attempt of nature to relieve an obstructed venous circulation, and that the erosion is a surface from which the epithelium has been washed away by the discharge constantly flowing over it, then such a course of treatment is to be deemed not only irrational but most hurtful. A whole generation of physicians has been misled by the delusion of *chronic inflammation and ulceration* of the uterus, conditions which no one has yet been able to demonstrate on the dead body."

We regret that these chapters on local treatment may not receive the attention which they merit from every man who holds contrary opinions. Before such a man makes another caustic application to the uterus it is his duty to weigh the evidence contained in this book and be convinced that the hard contracting scar surface which must be substituted for the epithelial membrane before the supposed ulcer can be removed is not productive of lasting evil. We are convinced of the soundness of Emmet's views, that the caustic always results in destruction of the mucous follicles and frequently in hypertrophy of the uterus and disordered innervation and circulation in the pelvis.

Injection of the uterine cavity is permitted and under certain conditions enjoined if the canal be prepared by previous dilation to admit free return of the injected fluid, otherwise it is condemned as a dangerous procedure. Dilatation of the canal by tents is a subject to which the author has evidently given much attention. He fully exposes the advantages of their use and the dangers of their abuse.

The author's conservatism is expressed in the following quotation:

"We should have the fear of cellulitis always before us, in the treatment of these diseases: as common as this complication is from cold and from other causes, it has its origin quite as frequently in carelessness on the part of the practitioner. We should never introduce the probe, a sponge tent, or an application within the uterine cavity, if the slightest indication of cellulitis can be detected. Nor should we attempt to correct a displacement of the uterus as long as any tenderness attributable to inflammatory origin can be detected by the finger. The female organs of generations have been mercifully endowed with a degree of tolerance to injury not possessed by the male, and woman is thus protected that she may be able to bear the perils of gestation. But few however of the many physicians who undertake to treat these diseases fully realize that there is naturally a limit to this immunity. No portion of the body has suffered more in consequence of incapacity on the part of members of the profession. * * * * * * * Under the guise of surgery the uterus has been subjected to a degree of malpractice, which would not have been tolerated by any other portion of the body; its cavity has been, and is still made the receptacle for agents so destructive that no conscientious man would employ them for the treatment of diseases in any other portion of the body without a full appreciation of his responsibility. But I trust that we have already passed the heroic age. * * *"

These two chapters are eminently conservative and satisfactory. They are full of precepts and suggestions of great practical value, and deserve not carelessly to be read, but profoundly to be studied. The practitioner who fortunately follows them will stand on a decided vantage ground.

*Chapter IX, on Ovulation and Menstruation*, contains fifteen exhaustive tables of statistics drawn from the author's extensive observation, and must furnish much new material for generalization and speculation. The author regards the physiology of menstruation as yet far from being fully settled, but respecting its relation to the uterine mucous membrane, believes with Tyler

Smith and others, that this membrane is disintegrated and thrown off at every period.

*Chapter X treats of the Various Menstrual Irregularities*, as symptoms, not as diseases. Disordered innervation and circulation and faulty nutrition of the uterus, are regarded in general as the immediate causes of painful menstruation. Mechanical dysmenorrhœa from flexions and other causes of obstruction in the uterine canal is thought to be very much less common than the popular opinion would indicate. Division of the cervix therefore is seldom advised, and the various forms of dilatation are to be invoked rather for the purpose of changing the condition of circulation in the endometrial membrane and the adjacent tissues than for the purpose of overcoming any obstruction to the passage of blood through the canal. We believe the author to be right in this matter, that the effect of dilatation upon the membrane is to hasten by pressure its periodical fatty degeneration and disintegration, thereby to relieve congestion by hastening the flow which may become established as soon as this membrane is cast off. We know of no other work which contains so much that is reliable and valuable on the subject of abnormal changes in the menstrual flow.

*Chapter XI. Congenital Absence and Accidental Atresia of the Vagina; Mode of operating for Establishing the Canal and for Evacuating Retained Menstrual Blood.*

Dr. Emmet completes the vaginal canal and gives free exit to all retained menstrual fluid in one operation and washes out the uterine cavity with warm carbolized water for the prevention of blood poisoning. He also employs the glass vaginal dilator of Sims in the artificial vagina until the process of healing is complete. His own success and that of others who have employed the same method has been exceptionally good. This chapter is well illustrated by wood cuts and contains a tabular statement of the author's experience of twenty-two cases.

*Chapter XII, on Pelvic Hematocele*, includes three engravings and a full history of the literature of the subject.

*Chapter XIII. Diseases of the Pelvic Cellular Tissue.* Dr. Emmet stands far in advance of the time in his appreciation of the relations of cellulitis to the diseases of women.

He says: "This disease is by far the most important one with which woman is afflicted. Many of the disappointments and bad results so often complained of in the management of diseases of women, in general practice, may be attributed to the existence of unrecognized cellulitis. * * * * I am confident that the greatest advances yet to be made in this branch of surgery will be from the immediate study of the cellular tissue of the pelvis. We shall there find the key to many of the patological changes now treated as uterine disease. * * * * To illustrate, the circulation in a portion of the cellular tissue may become obstructed from some cause (such as cellulitis) with the effect of producing congestive hypertrophy of the uterus from partial stagnation. One of the first attempts of nature would be to relieve temporarily this condition by an increase of secretion from the mucous follicles. As this discharge continued to flow the epithelium would at length be lost, and what has hitherto been termed ulceration would be produced.

The statistical history of three hundred and three cases is given in a number of tables which present the subject in its great variety of relations. These tables, five in number, are among the most elaborate in the book.

*Chapters XIV, XV, XVI, XVII, XVIII* and *XIX* are mainly devoted to the various displacements of the uterus. Time and space forbid an adequate review of these chapters. They include numerous illustrations and a vast amount of statistical information. Division of the cervix for flexions, except in comparatively rare instances, intra-uterine stem pessaries, pessaries having external attachments, sponge pessaries and pessaries which press upon the anterior wall of the uterus are condemned as dangerous or useless.

The author's modification of Sims' operation for cystocele is the best ever devised, since it not only replaces the prolapsed anterior vaginal wall, but also causes the uterus to be held temporarily in position until perineal support can be gained by the restoration of the perineal body which is almost invariably wanting in such cases.

*Chapter XX. Laceration of the Perineum.* The author's original contributions on this subject are for the first time given to the profession in book form. His operation of perineorrhaphy in which the rectocele is used as material of which to form the new perineal body instead of making a separate operation for its removal, and his method of passing the sutures for the restoration of the sphincter ani muscle, may be regarded as perfect.

*Chapter XXI. Inversion of the Uterus.*

*Chapter XXII. Subinvolution.*

*Chapters XXIII and XXIV. Laceration of the Cervix Uteri.* These chapters are entirely original and exhaustive. They are destined to revolutionize the entire pathology and treatment of so-called hypertrophy, elongation and ulceration of the cervix. We are so impressed with the vast importance of this subject that we make the following somewhat lengthy quotation :

"As soon as the practitioner becomes able to recognize this condition under its different forms, he will be surprised to find a new explanation of all his cases of elongated or hypertrophied cervix as well as those of ulceration. Let him in all such cases simply make the attempt with a tenaculum in each hand to bring the points A and B (fig. 90) together at C, and a revelation will be opened to him. It will be necessary to employ Sims' speculum or some other instrument of the same kind, for otherwise the condition will not be detected. This I believe to be difficult with any valvular or cylindrical speculum, for these put the parts on the stretch. To this fact is doubtless due the difference of opinion which exists to-day as to the frequency of this injury. But let any one once master the diagnosis and he will not fail to recognize the protean nature of laceration and will never see another ease of hypertrophied cervix or so-called elongated neck; moreover he will never have occasion afterward to amputate the cervix or any portion of it, except for malignant disease. This has been my experience during the past nine or ten years and in so large a practice, that, if hypertrophy and elongation existed I could not have failed to have recognized them. What observer has ever met with either of these conditions except after childbirth or abortion? * * * * I deny that such lesions exist. * * * * *I can but denounce amputation with scissors, knife or cautery of a so-called hypertrophy or elongation of the cervix as malpractice. I also deprecate as even more uncalled for the application of the cautery or caustics to heal a so-called ulceration on surfaces which can be readily united and brought into a healthy condition*" (by suture.)

Since the invention of Sims' speculum this is perhaps the greatest advance in gynæcology, both in the extent of its application, and in the beneficence in its results.

*Chapter XXV. Amputation of the Cervix Uteri.* The author declares that this operation is never called for except in malignant disease, and says that since he has appreciated the true pathology of what he once supposed to be hypertrophy and elongation, he has had no occasion to perform this operation for the relief of these conditions. The operation is characterized as productive of more unnecessary evil, and therefore as being to a greater extent a malpractice than any other procedure in uterine surgery. We await with impatience the day when these views may become general.

*Chapter XXVI. Cancer of the Uterus, Vagina, Rectum and External Organs of Generation.* Considerable evidence is given which points to laceration of the cervix as an exciting cause of epithelioma. The recent observations of Breisky and Veit, in Germany, tend to corroborate this view.

*Chapters XXVII, XXVIII and XXIX* are given to the subject of *Fibrous Growths of the Uterus.* The history, etiology and diagnosis and general management are satisfactorily presented unless exception be taken to the author's estimate of ergot as a means of radical cure. He bases his opinions upon his own and the observations of Hilderbrandt, of Königsberg.

"From the injudicious use of ergot in large quantities, much harm has resulted. * * * * As a rule, great benefit follows its use when administered in small and continued doses * * * * with the view of exciting only moderate contraction. Ergot should never be given in large doses until after the uterine canal has been dilated and until it be found that the tumor projects sufficiently to warrant the belief that it may become pedunculated by uterine contraction. I have committed this error myself, and have likewise frequently observed it in the practice of others. Should a tumor be found buried in the uterine wall, or so situated that it cannot become pedunculated, large doses of ergot can certainly accomplish no good. But on the contrary, if the uterus be excited to violent contraction without a purpose as it were, an increased amount of blood will naturally flow to the parts,often with the direct effect of causing cellulitis and even peritonitis. * * * A favorable condition for increasing the growth of the tumor."

In direct contrast with the above, is the evidence presented in the memoirs of Dr. Wm. H. Byford, of this city, who reports a number of cases which he regarded as intramural, and which therefore could not be made to pedunculate by uterine contraction, but which appear to have sloughed away under the influence of large and continued doses of ergot.

In a future edition we should be glad to see the ergot treatment more fully considered. The questions are: *Were some of the fibroids reported by Dr. Byford intra-mural, as he believes, or only submucous? May the ergot be relied upon generally to expel or starve out intra-mural fibroids? How great are the dangers from cellulitis, peritonitis, and from the blood poisoning which must accompany a sloughing fibroid of the uterus?*

The most important of Dr. Emmet's contributions to this subject, is his method of removing submucous fibroids by traction. It is a matter of surprise and regret that so rational a procedure should not have received more attention from previous writers, since it was given to the profession in a memoir several years ago.

*Chapter XXX. Diseases of the External Organs of Generation, Cervix and Uterine Canal.* The author's ideas of the injurious effects of cicatricial tissue on the cervix resulting from severe caustic applications, or from any other cause, is worthy of more than passing attention. The curette forceps and the dull wire curette are substituted for the sharp curette, which is discarded as dangerous.

*Chapters XXXI, XXXII and XXXIII. Vesico-Vaginal and Recto-Vaginal Fistula.* No other book contains so much of value relative to vesico-vaginal and recto-vaginal fistula, not excepting the author's own work on vesico-vaginal fistula, published several years ago.

*Chapter XXXIV. Diseases of the Urethra.*

*Chapter XXXV. Cystitis, Stone in the Bladder, and Ureters.* The subjects of vesical and urethral disease are admirably and, in many respects, originally treated. It is shown that two or three per cent. of all cases in which forcible dilation of the female urethra is practiced, result in permanent incontinence of urine, and the operation is therefore totally condemned, and artificial vesico-vaginal fistula is substituted as entirely safe and in cystitis more satisfactory.

The eight remaining chapters, including about one hundred pages, are given to diseases of the ovaries, and to ovarian and abdominal tumors.

An essential feature of the book will be found in the marvelous amount of its statistic and tabular information which offers a wide field for speculation, for reflection and for classification of the laws of disease.

The literary execution of the work may not be quite up to the standard of its scientific excellence, but the author's meaning is generally clear and a spirit of candor pervades every page.

In novelty and utility its equal is not to be found in the medical and surgical history of America, or in the gynæcological history of the world.

E. C. D.

ARTICLE XIX.—PARESIS OF THE SYMPATHETIC CENTERS FROM OVER EXCITATION BY HIGH SOLAR HEAT LONG CONTINUED AND SUDDENLY WITHDRAWN, ETC., SO-CALLED MALARIA, ITS ETIOLOGY, PATHOGENESIS, PATHOLOGY AND TREATMENT. By Chas. T. Reber, M.D. St. Louis: Geo. O. Rumbold & Co., 1879.

Moralists tell us all things have their use in this world, which all things must include books. The one now before us we imagine has already accomplished its mission. It has probably given pleasure to the author, work to the compositor, printer and binder; but any further service that it can contribute to the world lies beyond the ken of our short-sighted vision. Altogether it is 112 pages, and a very large part of it is made up of extracts. The rest is simply generalized statements and illogical assumptions.

In his chapter on "Animal Temperature," etc., the author makes the statement, "Some birds have a normal temperature of 112° F.," whereas Dr. E. Seguin, as far as we know, an authority on temperature, gives the maximum temperature of the guinea fowl and common duck, the highest temperature given by his tables, as 43.90° C. or 111° F.; only one degree less it is true, but "an inch is a great deal on the end of a man's nose." Then "Horses and cattle have a lower temperature than man." Looking again to E. Seguin's tables we find the French horse 36.80° C. or within .24° of 98° F., which our author gives as the normal human temperature; while the Arabian horse furnishes a temperature of 37.50° C. or 1.5° F. higher than the human normal of our author. The temperature of the ox is given by Dr. E. Seguin as identical with the Arabian horse, while the sheep varies from .23° C. lower to 3° higher than the Arabian horse. It is true the author's term "cattle" may embrace a wider field than the horse, ox and sheep, but it so happens that in the very extensive table of Dr. E. S. the French horse is the lowest tempered on the list.

We were favorably struck with the title of this book, but greatly disappointed on investigating its pages. We do not think it worth while to give a summation of the contents, for as we understand the object of review is to furnish subscribers some idea of the nature of a work more definite than can be obtained from the title, etc., and we think that is better accomplished by exhibiting the way in which one idea is handled; for when impartially selected the old classical proverb, "from one you may judge the whole," is peculiarly applicable. The author closes his book with the magnanimous sneer at those who are diligently laborious in the cause of positive knowledge. We were about to say that there is nothing worthy of note in the book, but there is, the author would have us discard the word "*malaria*" and substitute it by the term "*hyper-therma.*"

